# The Potential Contributions of Lethal and Edema Toxins to the Pathogenesis of Anthrax Associated Shock

**DOI:** 10.3390/toxins3091185

**Published:** 2011-09-20

**Authors:** Caitlin W. Hicks, Xizhong Cui, Daniel A. Sweeney, Yan Li, Amisha Barochia, Peter Q. Eichacker

**Affiliations:** 1 Cleveland Clinic Lerner College of Medicine, Cleveland, OH 44195, USA; Email: caitlin.hicks@gmail.com; 2 Howard Hughes Medical Institute-National Institutes of Health Research Scholar, National Institutes of Health, Bethesda, MD 20814, USA; 3 Critical Care Medicine Department, Clinical Center, National Institutes of Health, Bethesda, MD 20892, USA; Email: cxizhong@mail.cc.nih.gov (X.C.); yli@mail.cc.nih.gov (Y.L.); braochiaav@mail.cc.nih.gov (A.B.); 4 Medical Intensivist Program, Washington Hospital, Fremont, CA 94538, USA; Email: danielasweeney@yahoo.com

**Keywords:** anthrax, lethal toxin, edema toxin, shock, myocardial function

## Abstract

Outbreaks of *Bacillus anthracis* in the US and Europe over the past 10 years have emphasized the health threat this lethal bacteria poses even for developed parts of the world. In contrast to cutaneous anthrax, inhalational disease in the US during the 2001 outbreaks and the newly identified injectional drug use form of disease in the UK and Germany have been associated with relatively high mortality rates. One notable aspect of these cases has been the difficulty in supporting patients once shock has developed. Anthrax bacilli produce several different components which likely contribute to this shock. Growing evidence indicates that both major anthrax toxins may produce substantial cardiovascular dysfunction. Lethal toxin (LT) can alter peripheral vascular function; it also has direct myocardial depressant effects. Edema toxin (ET) may have even more pronounced peripheral vascular effects than LT, including the ability to interfere with the actions of conventional vasopressors. Additionally, ET also appears capable of interfering with renal sodium and water retention. Importantly, the two toxins exert their actions via quite different mechanisms and therefore have the potential to worsen shock and outcome in an additive fashion. Finally, both toxins have the ability to inhibit host defense and microbial clearance, possibly contributing to the very high bacterial loads noted in patients dying with anthrax. This last point is clinically relevant since emerging data has begun to implicate other bacterial components such as anthrax cell wall in the shock and organ injury observed with infection. Taken together, accumulating evidence regarding the potential contribution of LT and ET to anthrax-associated shock supports efforts to develop adjunctive therapies that target both toxins in patients with progressive shock.

## 1. Introduction

Although the cutaneous form of *Bacillus anthracis* infection is most common worldwide, inhalational and gastrointestinal anthrax, as well as the recently recognized soft tissue infection in injection drug users, termed injectional anthrax, are more lethal [1,2,3]. Death in these latter three is almost always preceded by progressive hemodynamic instability and shock. Compared to more commonly encountered types of bacteria, shock with anthrax appears to be potentially more resistant to conventional types of hemodynamic support. In the 2001 US outbreak of anthrax, all patients who developed shock died despite aggressive intensive care unit support. While the number of patients involved in this outbreak was small, a mortality rate of 100% is much greater than typically reported for other types of treated bacterial shock [4]. Similarly, patients who developed shock in the recent UK anthrax outbreak among injection drug users have also been reported to be more difficult to manage with standard hemodynamic support [5].

While *B. anthracis* produces several components which may play an important role in shock, its two exotoxins, lethal toxin (LT) and edema toxin (ET), have received particular attention. Past and recent work directed at the cardiovascular effects of LT and ET provides important insights into the pathogenesis and potential management of shock with anthrax. Here, we review some of that work, and also briefly consider other components of *B. anthracis* that might contribute to shock. 

## 2. Lethal and Edema Toxin Structure and Intracellular Effects

Lethal and edema toxins are A-B type exotoxins composed of 2 proteins each, the A component being either lethal factor (LF) and/or edema factor (EF), respectively, and the B component, common to both, being protective antigen (PA). Following infection, PA released into the circulation binds to host cell surface receptors such as tumor endothelial marker 8 (TEM8) or capillary morphogenesis gene 2 (CMG2) [6,7,8,9]. Possibly most highly expressed in endothelial cells, both receptors have been demonstrated in a range of tissues including heart, lung, small intestine, spleen liver, kidney, skeletal muscle, and skin [10,11]. 

By itself, PA does not appear to have pathologic effects on the host [12]; its importance lies in its ability to facilitate intracellular delivery of the toxic anthrax factors (LF and EF). Upon binding to receptors, the 83 kD PA molecules undergo furin cleavage into 20 kD portions (freed into extracellular space), and 63 kD subunits that remain cell surface bound and combine to produce heptamers [13,14]. One to three LF and/or EF subunits competitively bind to these heptamers forming complexes, which undergo endocytosis and progressive acidification, after which EF and LF are released intracellularly [15]. 

Lethal factor is a zinc metalloprotease that inactivates mitogen-activated protein kinase kinases (MAPKK) 1–4, 6 and 7. While LF has been demonstrated to cause lysis of macrophages *in vitro* [6,16] and inhibit important host cell functions (e.g., innate and adaptive immunity and apoptosis), how it contributes to death associated with LT is still unclear. Edema factor is a calmodulin-dependent adenyl cyclase that increases intracellular cAMP concentration and impairs host defenses, including inhibition of phagocytosis [6,17,18]. It causes edema when injected subcutaneously into experimental animals [19]. Recent reviews as well as other contributions in this issue provide comprehensive overviews of the likely intracellular effects of LF and EF [16,18,20].

While older studies suggested that LT was the key virulence factor required for the lethal effects of *B.* anthracis, more recent studies have emphasized the potential importance of ET [12,21,22,23]. Both LT and ET are capable of producing cardiovascular effects leading to shock, and given their different actions, the combination of both toxins in active infection has potentially additive effects on increasing the severity of anthrax-associated shock. 

## 3. Lethal Toxin

Lethal toxin has been a focus of research since its original description as a bacterial component closely associated with death due to anthrax [24,25]. Shortly after the original description of LT in the 1950’s, a small number of studies in guinea pigs, rats and rhesus monkeys administered LT as a bolus, suggested that this toxin caused extravasation of fluid, hemoconcentration and shock [26,27,28,29]. However, based on limited measures of cardiac and pulmonary function, it was concluded that this shock was a terminal event related in part to respiratory failure [8]. Over the 30 years following these early investigations, no additional studies characterizing the cardiopulmonary effects of LT were reported. However, in contrast to findings from these early studies with LT, during the 2001 US anthrax outbreak, shock occurred well before death in non-survivors and did not initially appear to result from respiratory failure [1]. Similarly, shock has been a primary event noted in the UK anthrax outbreak in injection drug users [5].

**Figure 1:**
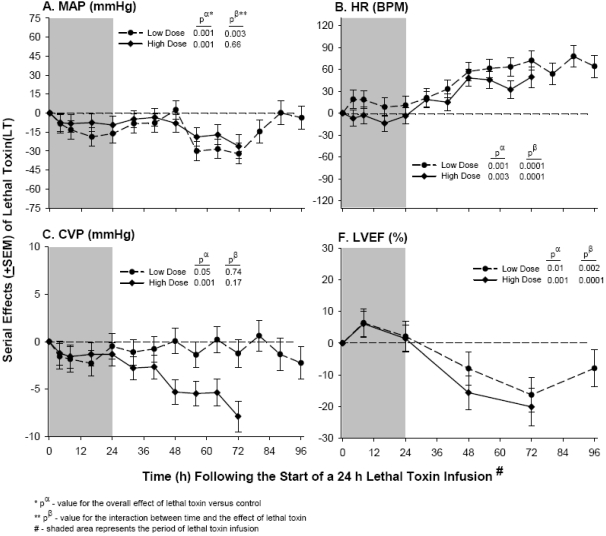
Continuously sedated and mechanically ventilated canines with indwelling systemic and pulmonary arterial catheters were challenged with 24 h infusions of low or high doses of lethal toxin (LT) or diluent only (controls) [23]. Panels A through D show the serial mean (±SEM) effects compared to controls of the two doses of LT on changes from baseline for mean arterial blood pressure (MAP, mmHg), heart rate (HR, bpm), central venous pressure (CVP, mmHg), and left ventricular ejection fraction (LVEF, %). The shaded gray area denotes the 24 hour toxin infusion period. Increases or decreases with toxin compared to controls are denoted by symbols above or below the dashed horizontal no effect line, respectively. The p statistics are shown in each panel for the overall effect of toxin compared to control (p^α^) and the interaction between time and the effect of toxin (p^β^). For statistical analysis serial changes from baseline with LT were compared to serial changes from baseline in controls. However, for clarity in this figure the serial effects of challenge (*i.e.*, toxin minus control) are shown. Overall, MAP, CVP and LVEF decreased while HR increased with LT compared to controls.

*In vivo* studies from several laboratories over the past 5 to 10 years, while employing differing species, toxin challenges and methods of measure, together suggest that LT does indeed produce shock and organ injury directly, and that the underlying physiologic mechanisms are likely multifactorial. Based on the pattern of histological injury noted in mice challenged with LT as an intraperitoneal bolus, Moayeri *et al.* concluded that lethality with LT was related to tissue hypoxia such as would occur with organ hypoperfusion [30]. In rats with indwelling central venous and arterial catheters, we found that 24 h LT infusions produced progressive hypotension that was significantly greater in animals that later died [31]. These changes were associated with evidence of hemoconcentration (*i.e.*, increased hemoglobin concentrations) and tissue hypoperfusion (*i.e.*, increased lactate levels). In subsequent studies with sedated and mechanically ventilated canines with invasive hemodynamic monitoring, 24-h LT infusions also caused progressive shock and reductions in central venous pressure over the subsequent 72 h observation period (Figure 1) [23]. These findings are consistent with a potential effect of LT on the peripheral vasculature; several groups have reported that LT can disrupt endothelial barrier function. Stimulation of endothelial cell apoptosis, alteration of actin fibers and cadherins and mast cell activation are invoked as possible mechanisms causing endothelial dysfunction from *in vitro* experiments [32,33,34,35,36]. In addition, work in a zebrafish model has implicated LT disruption of MAPKKs in endothelial dysfunction [37,38]. The time course of endothelial changes from *in vitro* studies is consistent with the gradual onset of clinical changes noted in the canine model. Interestingly though, in rat and canine models, LT challenge was not associated with pleural effusions like those noted in humans with anthrax infection [1]. This finding suggests that other factors, possibly working in combination with LT, are necessary for this manifestation of active infection. 

In addition to its potential effects on the peripheral vasculature, there is evidence to suggest that LT may directly depress myocardial function. We found that in sedated, mechanically ventilated canines, progressive hypotension occurred, and left ventricular ejection fraction (LVEF) measured with echocardiography also progressively decreased from 48 to 96 h after initiating a 24 h LT infusion in doses producing low (*n* = 6) or high (*n* = 9) lethality rates compared to a control group (*n* = 9) (Figure 1) [23]. Pulmonary artery occlusion pressure was not reduced, suggesting that decreases in preload were not the primary basis for hypotension or reduced LVEF. Heart rate did increase over time, and cardiac output was maintained (Figure 1). Other labs have also noted myocardial effects of LT. Watson *et al* assessed myocardial function with echocardiography in rats administered injections of LT producing hypotension and lethality [39,40]. They noted that 1–2 h after challenge, LT increased velocity of propagation and left ventricular diastolic and systolic areas [39]. In another report, the same authors noted that by 18 h after challenge, LT also increased left ventricular systolic area and decreased velocity of propagation, circumferential fiber shortening and left ventricular ejection fraction [40]. In a subsequent abstract and review, the group went on to describe an experiment employing pressure-volume loop measures in canines where, compared to controls, LT injection in 2 animals was associated with reduced stroke volume, end systolic pressure and ejection fraction, and increased left ventricular end diastolic pressure (LVEDP), in an overall pattern consistent with severe heart failure by 96 h after challenge [41]. Consistent with these findings, Moayeri *et al.* reported that intraperitoneal LT challenge in mice was associated with reduced LVEF and myocardial changes on electron microscopy as early as 6 h [42]. Although the mechanisms underlying the potential myocardial effects of LT are not clear, inhibition of MAPK ERK1/2 has been shown to produce stress-induced apoptosis and heart failure, whereas augmentation is protective [43,44,45]. Also, a recent *in vitro* study has suggested that LT may depress cardiomyocyte function via an NADPH oxidase-dependent mechanism [46]. 

Despite the cardiac effects of LT noted in *in vivo* models, using an isolated perfused rat heart system observed over a 4 h period we were only able to document a direct effect of LT on myocardial function with toxin doses substantially higher than those producing shock and lethality *in vivo* [22]. This, in combination with the *in vivo* studies, suggests that the effects of LT on myocardial function may take at the least several hours to develop. It is also possible that peripheral vascular changes and resulting hypotension may aggravate myocardial changes seen in *in vivo* models. In our canine model, although pulmonary arterial occlusion pressure (PAOP) was not reduced, central venous pressure (CVP) was, and fluid loading increased LVEF to some degree [23]. 

**Figure 2:**
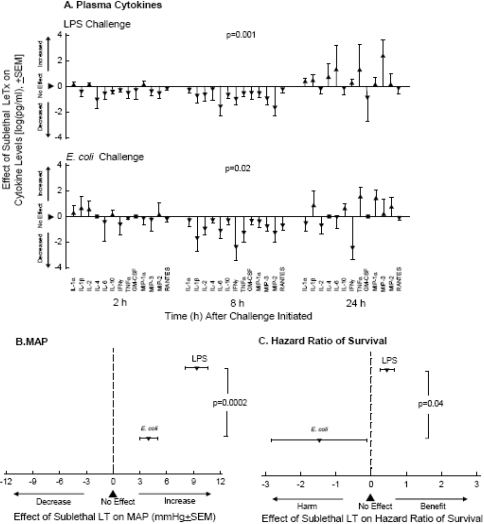
Effects of sublethal doses of lethal toxin (LT) compared to placebo administered 3 h before intravenous lipopolysaccharide (LPS) or intratracheal *E. coli* challenge on plasma cytokine levels (panel A), mean arterial blood pressure (MAP, panel B) and the hazard ratio of survival (panel C) [47]. Cytokines were measured at 2, 8 and 24 h; blood pressure was averaged over the 24 h after challenge; and survival was assessed at 168 h. With both LPS and *E. coli*, LT had variable effects on cytokines at 2 and 24 h but uniformly reduced all 13 at 8 h. The consistent decreases caused by LT in all cytokines at 8 h were significantly different from the more variable changes noted at 2 and 24 h with both challenges (*p* = 0.001 and 0.02 as shown in Panel A). These anti-inflammatory effects of sublethal LT were associated with higher than control MAP and survival with LPS challenge. However, with *E. coli* the effects were significantly different; increases in MAP were smaller and survival was actually reduced.

Finally, LT’s contribution to the development of shock may also involve its apparent immunosuppressive effects. Cardiovascular dysfunction during severe bacterial infection is thought to relate in part to the damaging effects of inflammatory mediators (e.g., cytokines, nitric oxide, oxygen free radicals) produced by the host during the innate immune response. One report suggested that macrophage damage by LT might produce increased circulating inflammatory cytokine levels, and that macrophage inhibition was beneficial with lethal doses of LT [48]. Subsequent studies however have not found a clear association between the cardiovascular dysfunction produced by LT and excessive inflammatory mediator production [8,12,30,31]. Such findings are consistent with LF’s known effect on suppressing stress kinase pathways, which are typically important contributors to the inflammatory response during infection [16,20,49]. In fact, we found in a rat model that pretreatment of animals with a sub-lethal dose of LT actually blunted the inflammatory response stimulated by either lipopolysaccharide (LPS) or intratracheal *E. coli* challenges (Figure 2) [47]. Notably however, while pretreatment with LT increased survival with LPS, it reduced survival with *E. coli*. Therefore, as has been suggested by other investigators, LT may also participate in the pathogenesis of shock and organ injury during anthrax infection by suppressing host microbial clearance and contributing to the very high bacterial loads noted in subjects dying with infection [49]. 

## 4. Edema Toxin

ET has received considerably less attention than LT since mutant deletion studies as well as direct comparisons of the two toxins suggested that the latter played a more central role in anthrax pathogenesis [50]. A study in rabbits suggested that LT may be produced in greater proportion than ET during active infection [51]. We reported that when administered as 24 h infusions, on a molar dose basis, LT was 5 to 20 fold more lethal than ET in both rats and canines [12,23]. In addition, efforts to generate large quantities of purified EF from *B. anthracis* cultures proved difficult, resulting in low yields and high levels of contaminating proteins [52]. Despite such data, there are still reasons to believe that ET plays an important role in the pathogenesis of shock during anthrax. Furthermore, recent developments allowing the generation of recombinant EF from cultures of *E. coli* have demonstrated consistently large quantities of >97% pure EF [53], mitigating previous concerns that the degree of purity of some edema factor preparations may skew the interpretation of cell culture or *in vivo* data.

From its first description and as its name implies, ET was noted for its ability to promote local extravasation of fluid [25]. Clinically, the notable extravascular fluid collections, such as the pleural effusions observed during the 2001 US outbreak, suggested that ET might play an important role in systemic infection [54]. Some patients in the recent UK outbreak also demonstrated striking soft tissue edema, not only locally at the sites of initial infection, but at more distant sites as well [5]. Finally, we noted that mortality from LT challenge increased in both rat and canine models when LT was co-administered with equimolar but nonlethal doses of ET [12,23]. 

**Figure 3:**
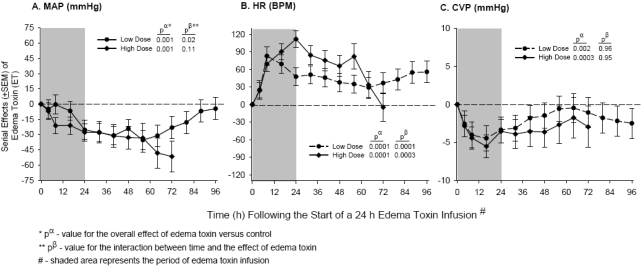
Continuously sedated and mechanically ventilated canines with indwelling systemic and pulmonary arterial catheters were challenged with 24 h infusions of low or high doses of edema toxin (ET) or diluent alone (controls) [23]. Panels A through C show the serial mean (±SEM) effects compared to controls of the two doses of ET on changes from baseline for mean arterial blood pressure (MAP, mmHg), heart rate (HR, bpm), and central venous pressure (CVP, mmHg). The format and presentation of data is similar to Figure 1. Challenge with 24 h ET infusions in canines using both high and low doses of toxin produced rapid reductions in both central venous and arterial blood pressure and increases in heart rate that persisted for up to 72 h after challenge.

While the mechanisms underlying shock with LT appear to be multifactorial and thus have been difficult to delineate, those underlying ET’s possible role may be easier to understand. ET has potent calmodulin dependent adenylcyclase activity, estimated to be 1000 times greater than that associated with eukaryotic adenylcyclases [17]. *In vitro*, ET increases intracellular cAMP to very high levels; cAMP is a key in mediator of vasodilation of both arteries and veins [55,56]. We noted rapid reductions in both central venous and arterial blood pressure in canines in patterns that persisted for up to 72 h after challenge with 24 h ET infusions, using doses causing high or low mortality (Figure 3) [23]. In a rat model, non-lethal and lethal ET infusions also produced rapid, persistent reductions in blood pressure [12]. Notably in both rat and canine studies, ET infusion was not associated with evidence of hemoconcentration (*i.e.*, circulating hemoglobin concentrations were not increased) or extravascular fluid collections (e.g., pleural effusions or ascites), suggesting that reductions in central venous pressure (CVP) might in part represent relaxation of venous capacitance vessels. In both models, ET caused tachycardia that appeared to be out of proportion to other hemodynamic changes, but was consistent with increased cAMP in cardiac pacemaker cells (Figure 3) [57,58]. Echocardiography also demonstrated evidence of reduced preload in rats administered a rapid ET challenge by other investigators, although no extravasation of fluid was noted on lung pathology.

**Figure 4:**
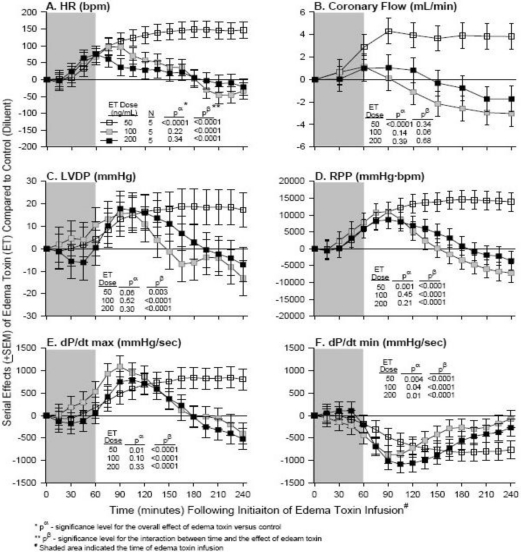
The difference in change from baseline from controls (no toxin) is shown as serial effects (mean ± SEM) of three doses of edema toxin (ET, 50, 100, and 200 ng/mL) on heart rate (HR), coronary flow, left ventricular developed pressure (LVDP), rate pressure product (RPP), and left ventricular dP/dt max and dP/dt min in a non-recirculating isolated rat heart model [22]. The shaded area represents when toxin was administered. The p statistics are shown for the overall effects of toxin versus control (p^α^), and the interaction between these effects and time (p^β^). For statistical analysis serial changes from baseline with edema toxin were compared to serial changes from baseline in controls. However, for clarity in this figure, the serial effects of challenge (*i.e.*, toxin minus control) are shown. In the model, ET challenge produced significant tachycardia, caused substantial increases in coronary flow rate consistent with a direct vasodilatory effect of the toxin, and had inotropic effects that were transient with higher ET doses but persistent at lower ones.

**Figure 5:**
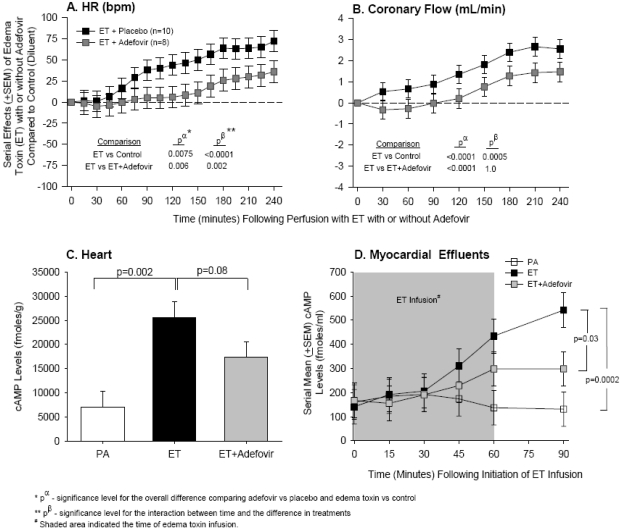
Panels A and B show the serial effects (mean ± SEM) of edema toxin (ET) with or without adefovir compared to controls on heart rate (HR) (Panel A) and coronary flow (Panel B) in a recirculating, constant pressure, isolated, perfused rat heart model [22]. Hearts were exposed to toxin and treatment throughout the perfusion period. The p statistics are shown for the overall effects of toxin with placebo versus with adefovir compared to control (no toxin) (p^α^), and the interaction between these effects and time (p^β^). For statistical analysis serial changes from baseline with edema toxin with or without adefovir were compared to serial changes from baseline in controls. However, for clarity in this figure the serial effects of edema toxin alone or with treatment (*i.e.*, toxin with or without adefovir minus control) are shown. Panel C shows mean (±SEM) cAMP levels in myocardial tissue 30 min after completion of an hour of perfusion with PA alone, edema toxin (ET) alone, or edema toxin with adefovir. Panel D shows serial mean (±SEM) cAMP levels in effluent from hearts perfused for 60 min with PA alone, ET alone, or ET with adefovir. The shaded area denotes the time of toxin infusion. Consistent with its predominant mechanism of action, ET produced increases in both myocardial tissue and effluent cAMP levels that were inhibited by adefovir, a nucleoside agent which has been shown to inhibit EF adenylcyclase activity *in vitro.*

In a perfused rat heart model that is free of preload and after-load influences, we found that ET challenge, in addition to producing significant tachycardia, caused substantial increases in coronary flow rate consistent with a direct vasodilatory effect of the toxin [22]. Interestingly, in this isolated system, ET also had inotropic effects (Figure 4). While these effects were transient with higher ET doses, they persisted at lower ones. Consistent with its predominant mechanism of action, ET produced increases in both myocardial tissue and effluent cAMP levels (Figure 5). Adefovir, a nucleoside agent which has been shown to inhibit EF adenylcyclase activity *in vitro* [59], inhibited ET associated increases in heart rate (HR), coronary flow and cAMP levels (Figure 5). Given its effect on intracellular cAMP, ET may be a potential cause of the vasopressor resistant shock noted in patients in both the U.S. and U.K. outbreaks [5], since such agents can blunt the vasoconstrictive effects of agents like norepinephrine and phenylephrine. 

**Figure 6:**
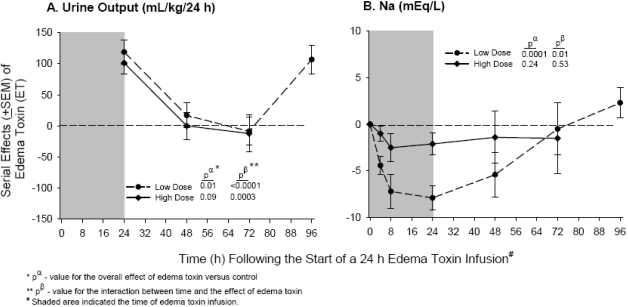
Continuously sedated and mechanically ventilated canines with indwelling systemic and pulmonary arterial catheters were challenged with 24 h infusions of low or high doses of edema toxin (ET) or diluent alone (controls) [23]. Panels A and B, respectively, show the serial mean (±SEM) effects compared to controls of the two doses of ET on 24 h rates of urine output (mL/kg/24 h) and on the changes from baseline in serum sodium (Na) levels. The format and presentation of data is similar to Figure 1. Despite the development of shock in the model, ET challenge resulted in ten-fold increases in urine output and decreased serum sodium, suggesting that inappropriate sodium and water losses may aggravate shock in patients with anthrax.

Additionally, we noted in our canine model that even as shock developed, ET challenge resulted in ten-fold increases in urine output, along with decreased serum sodium (Figure 6) [23]. Since increased tubular cAMP stimulated by other types of toxins (e.g., cholera toxin) is associated with inappropriate sodium and water losses, it is possible that similar mechanisms may aggravate shock in patients with anthrax [60,61,62]. Of note, hyponatremia has been a frequently reported abnormality in patients presenting with anthrax [63]. Furthermore, in a murine model, ET was associated with adrenal gland necrosis, which could also aggravate sodium and water losses [21].

Like LT, ET may also have immunosuppressive effects. Challenge with ET alone does not stimulate the typical inflammatory mediator release noted with most types of bacterial infection [47]. Furthermore, ET has been shown to alter several different aspects of host defense, including reduced activation and function of antigen-presenting cells, increased release of cytokines from dendritic cells, and impaired chemotaxis and differentiation of T lymphocytes [49]. 

Although on a molar dose basis ET is less lethal than LT and the production of LT may be greater than ET during infection, the two toxins together may have synergistic effects [12,51]. In mouse, rat and canine models, nonlethal ET doses have been shown to add to the lethality of LT dose [12,23,64]. The basis for such synergy is not clear. ET has been shown to up-regulate the expression of PA receptors on macrophages and dendritic cells *in vitro*, thereby increasing the rate of toxin internalization [65]. ET has also been shown to potentiate the inhibitory effects of LT on T-cell and dendritic cell function and chemotaxis [66,67].

## 5. Other Components that May Aggravate Toxin Associated Shock

Although LT and ET have been the focus of research as pathogenic products of *B. anthracis*, other components of this organism such as bacterial cell wall and several metalloproteinases may also be important. In contrast to lethal or shock-inducing doses of LT or ET, challenge with live *B. anthracis* organisms does stimulate inflammatory mediator release [68,69,70,71]. As with other types of sepsis, this inflammation may contribute to shock and organ injury with anthrax. 

There is evidence suggesting that *B. anthracis* cell wall may stimulate this inflammatory response. Human peripheral blood mononuclear cells, possibly via stimulation of TLR2/6 heterodimers, release TNFα, IL-1β and IL-6 in response to challenge with whole *B. anthracis* cell wall [72,73]. Anthrax infection *in vitro* also results in NOD-2 dependent IL-1β release [74]. We noted dose dependent increases in lethality, lactate, circulating inflammatory cytokine, chemokine and nitric oxide levels and thrombocytopenia when we challenged rats with whole anthrax cell wall [75]. Peptidoglycan appears to be the component of the cell wall largely responsible for its immuno-stimulatory effects, and in fact, peptidoglycan is shed by replicating bacteria [76,77]. This ability of anthrax cell wall to produce a robust intravascular inflammatory response and participate in the pathogenesis of shock could be important; patients and animals dying with anthrax have very high bacterial loads providing a reservoir of active cell wall constituents [73,77]. 

Besides LF, *B. anthracis* also produces proteases that may be important in anthrax infection. The delta Ames (pXO1^−^ and pXO2^−^) anthrax strain produced metalloproteases belonging to the M4 thermolysin and M9 bacterial collagenase families in culture studies [78]. Mice develop hemorrhagic tissue injury in response to purified preparations of these proteases. Moreover, animals challenged with Sterne strain spores showed better survival if treated with chemical inhibitors or immune serum against the M4 and M9 proteases of *B. anthracis* [78]. Additional studies of homologue proteases have implicated them in other pathophysiologic effects, including the interruption of tight junctions in *Vibrio cholerae* infections, and the cleavage of antitrypsin-1α, TNF-α and IL-2 on human T cell surfaces in Legionella infections [79,80]. 

## 6. Conclusions

Both LT and ET have the ability to produce significant cardiovascular dysfunction at either the peripheral vascular or myocardial levels, or both. The ability of ET to also potentially interfere with renal sodium retention could aggravate the direct cardiovascular effects of either toxin. Importantly, both ET and LT likely exert their cardiovascular effects via very different and potentially additive mechanisms; some *in vivo* data suggests that such additive effects are indeed the case [12,23]. Therefore, utilization of adjunctive agents with the ability to inhibit the two toxins together may very well be needed in patients progressing to shock despite appropriate antibiotic therapy and aggressive hemodynamic support. Historically, antibodies to individual components of the bacteria were used even before antibiotic therapy was available [81]. Two PA directed antibody preparations, one polyclonal (Anthrax Immune Globulin, Cangene Corporation, Winnipeg, ME, Canada) and one monoclonal (Raxibacumab, Human Genome Sciences, Inc., Rockville, MD), have been made available in recent outbreaks or added to the U. S. Strategic National Stockpile [82,83,84,85]. However, while *in vitro* or *in vivo* data has supported the effectiveness of these agents, their clinical efficacy in humans must be clarified. A variety of other types of inhibitors have also been proposed [8,86]. Just as important as determining the effectiveness of adjunctive therapies directed against LT and ET is defining how other components of the bacteria may contribute to cardiovascular dysfunction during infection.

## References

[1] Jernigan D.B., Raghunathan P.L., Bell B.P., Brechner R., Bresnitz E.A., Butler J.C., Cetron M., Cohen M., Doyle T., Fischer M. (2002). Investigation of bioterrorism-related anthrax, United States, 2001: Epidemiologic findings. Emerg. Infect. Dis..

[2] Ramsay C.N., Stirling A., Smith J., Hawkins G., Brooks T., Hood J., Penrice G., Browning L.M., Ahmed S. (2010). An outbreak of infection with *Bacillus anthracis* in injecting drug users in Scotland. Euro. Surveill..

[3] Ringertz S.H., Hoiby E.A., Jensenius M., Maehlen J., Caugant D.A., Myklebust A., Fossum K. (2000). Injectional anthrax in a heroin skin-popper. Lancet.

[4] Winters B.D., Eberlein M., Leung J., Needham D.M., Pronovost P.J., Sevransky J.E. (2010). Long-term mortality and quality of life in sepsis: A systematic review. Crit. Care Med..

[5] Booth M.G., Hood J., Brooks T.J., Hart A. (2010). Anthrax infection in drug users. Lancet.

[6] Bradley K.A., Mogridge J., Mourez M., Collier R.J., Young J.A. (2001). Identification of the cellular receptor for anthrax toxin. Nature.

[7] Scobie H.M., Rainey G.J., Bradley K.A., Young J.A. (2003). Human capillary morphogenesis protein 2 functions as an anthrax toxin receptor. Proc. Natl. Acad. Sci. USA.

[8] Sherer K., Li Y., Cui X., Eichacker P.Q. (2007). Lethal and edema toxins in the pathogenesis of *Bacillus anthracis* septic shock: Implications for therapy. Am. J. Respir. Crit. Care Med..

[9] van der Goot G., Young J.A. (2009). Receptors of anthrax toxin and cell entry. Mol. Aspects. Med..

[10] Bell S.E., Mavila A., Salazar R., Bayless K.J., Kanagala S., Maxwell S.A., Davis G.E. (2001). Differential gene expression during capillary morphogenesis in 3D collagen matrices: Regulated expression of genes involved in basement membrane matrix assembly, cell cycle progression, cellular differentiation and G-protein signaling. J. Cell Sci..

[11] Carson-Walter E.B., Watkins D.N., Nanda A., Vogelstein B., Kinzler K.W., St Croix B. (2001). Cell surface tumor endothelial markers are conserved in mice and humans. Cancer Res..

[12] Cui X., Li Y., Li X., Laird M.W., Subramanian M., Moayeri M., Leppla S.H., Fitz Y., Su J., Sherer K. (2007). *Bacillus anthracis* edema and lethal toxin have different hemodynamic effects but function together to worsen shock and outcome in a rat model. J. Infect. Dis..

[13] Klimpel K.R., Molloy S.S., Thomas G., Leppla S.H. (1992). Anthrax toxin protective antigen is activated by a cell surface protease with the sequence specificity and catalytic properties of furin. Proc. Natl. Acad. Sci. USA.

[14] Milne J.C., Furlong D., Hanna P.C., Wall J.S., Collier R.J. (1994). Anthrax protective antigen forms oligomers during intoxication of mammalian cells. J. Biol. Chem..

[15] Mogridge J., Cunningham K., Collier R.J. (2002). Stoichiometry of anthrax toxin complexes. Biochemistry.

[16] Tonello F., Montecucco C. (2009). The anthrax lethal factor and its MAPK kinase-specific metalloprotease activity. Mol. Aspects. Med..

[17] Leppla S.H. (1982). Anthrax toxin edema factor: A bacterial adenylate cyclase that increases cyclic AMP concentrations of eukaryotic cells. Proc. Natl. Acad. Sci. USA.

[18] Tang W.J., Guo Q. (2009). The adenylyl cyclase activity of anthrax edema factor. Mol. Aspects. Med..

[19] Tippetts M.T., Robertson D.L. (1988). Molecular cloning and expression of the *Bacillus anthracis* edema factor toxin gene: A calmodulin-dependent adenylate cyclase. J. Bacteriol..

[20] Moayeri M., Leppla S.H. (2009). Cellular and systemic effects of anthrax lethal toxin and edema toxin. Mol. Aspects. Med..

[21] Firoved A.M., Miller G.F., Moayeri M., Kakkar R., Shen Y., Wiggins J.F., McNally E.M., Tang W.J., Leppla S.H. (2005). *Bacillus anthracis* edema toxin causes extensive tissue lesions and rapid lethality in mice. Am. J. Pathol..

[22] Hicks C.W., Li Y., Okugawa S., Solomon S.B., Moayeri M., Leppla S.H., Mohanty A., Subramanian G.M., Mignone T.S., Fitz Y. (2011). Anthrax edema toxin has cAMP-mediated stimulatory effects and high-dose lethal toxin has depressant effects in an isolated perfused rat heart model. Am. J. Physiol. Heart Circ. Physiol..

[23] Sweeney D.A., Cui X., Solomon S.B., Vitberg D.A., Migone T.S., Scher D., Danner R.L., Natanson C., Subramanian G.M., Eichacker P.Q. (2010). Anthrax lethal and edema toxins produce different patterns of cardiovascular and renal dysfunction and synergistically decrease survival in canines. J. Infect. Dis..

[24] Smith H., Keppie J., Stanley J.L., Harris-Smith P.W. (1955). The chemical basis of the virulence of *Bacillus anthracis*. IV. Secondary shock as the major factor in death of guinea-pigs from anthrax. Br. J. Exp. Pathol..

[25] Stanley J.L., Smith H. (1961). Purification of factor I and recognition of a third factor of the anthrax toxin. J. Gen. Microbiol..

[26] Beall F.A., Dalldorf F.G. (1966). The pathogenesis of the lethal effect of anthrax toxin in the rat. J. Infect. Dis..

[27] Bonventre P.F., Eckert N.J. (1963). Toxin production as a criterion for differentiating *Bacillus cereus* and *Bacillus anthracis*. J. Bacteriol..

[28] Fish D.C., Lincoln R.E. (1968). *In vivo*-produced anthrax toxin. J. Bacteriol..

[29] Vick J.A., Lincoln R.E., Klein F., Mahlandt B.G., Walker J.S., Fish D.C. (1968). Neurological and physiological responses of the primate to anthrax toxin. J. Infect. Dis..

[30] Moayeri M., Haines D., Young H.A., Leppla S.H. (2003). *Bacillus anthracis* lethal toxin induces TNF-alpha-independent hypoxia-mediated toxicity in mice. J. Clin. Invest..

[31] Cui X., Moayeri M., Li Y., Li X., Haley M., Fitz Y., Correa-Araujo R., Banks S.M., Leppla S.H., Eichacker P.Q. (2004). Lethality during continuous anthrax lethal toxin infusion is associated with circulatory shock but not inflammatory cytokine or nitric oxide release in rats. Am. J. Physiol. Regul. Integr. Comp. Physiol..

[32] Gozes Y., Moayeri M., Wiggins J.F., Leppla S.H. (2006). Anthrax lethal toxin induces ketotifen-sensitive intradermal vascular leakage in certain inbred mice. Infect. Immun..

[33] Kirby J.E. (2004). Anthrax lethal toxin induces human endothelial cell apoptosis. Infect. Immun..

[34] Rolando M., Munro P., Stefani C., Auberger P., Flatau G., Lemichez E. (2009). Injection of Staphylococcus aureus EDIN by the *Bacillus anthracis* protective antigen machinery induces vascular permeability. Infect. Immun..

[35] Warfel J.M., Steele A.D., D’Agnillo F. (2005). Anthrax lethal toxin induces endothelial barrier dysfunction. Am. J. Pathol..

[36] Guichard A., McGillivray S.M., Cruz-Moreno B., van Sorge N.M., Nizet V., Bier E. (2010). Anthrax toxins cooperatively inhibit endocytic recycling by the Rab11/Sec15 exocyst. Nature.

[37] Bolcome R.E., Chan J. (2010). Constitutive MEK1 activation rescues anthrax lethal toxin-induced vascular effects *in vivo*. Infect. Immun..

[38] Bolcome R.E., Sullivan S.E., Zeller R., Barker A.P., Collier R.J., Chan J. (2008). Anthrax lethal toxin induces cell death-independent permeability in zebrafish vasculature. Proc. Natl. Acad. Sci. USA.

[39] Watson L.E., Kuo S.R., Katki K., Dang T., Park S.K., Dostal D.E., Tang W.J., Leppla S.H., Frankel A.E. (2007). Anthrax toxins induce shock in rats by depressed cardiac ventricular function. PLoS One.

[40] Watson L.E., Mock J., Lal H., Lu G., Bourdeau R.W., Tang W.J., Leppla S.H., Dostal D.E., Frankel A.E. (2007). Lethal and edema toxins of anthrax induce distinct hemodynamic dysfunction. Front. Biosci..

[41] Golden H.B., Watson L.E., Lal H., Verma S.K., Foster D.M., Kuo S.R., Sharma A., Frankel A., Dostal D.E. (2009). Anthrax toxin: Pathologic effects on the cardiovascular system. Front. Biosci..

[42] Moayeri M., Crown D., Dorward D.W., Gardner D., Ward J.M., Li Y., Cui X., Eichacker P., Leppla S.H. (2009). The heart is an early target of anthrax lethal toxin in mice: A protective role for neuronal nitric oxide synthase (nNOS). PLoS Pathog..

[43] Lips D.J., Bueno O.F., Wilkins B.J., Purcell N.H., Kaiser R.A., Lorenz J.N., Voisin L., Saba-El-Leil M.K., Meloche S. (2004). MEK1-ERK2 signaling pathway protects myocardium from ischemic injury *in vivo*. Circulation.

[44] Purcell N.H., Wilkins B.J., York A., Saba-El-Leil M.K., Meloche S., Robbins J., Molkentin J.D. (2007). Genetic inhibition of cardiac ERK1/2 promotes stress-induced apoptosis and heart failure but has no effect on hypertrophy *in vivo*. Proc. Natl. Acad. Sci. USA.

[45] Yamaguchi O., Watanabe T., Nishida K., Kashiwase K., Higuchi Y., Takeda T., Hikoso S., Hirotani S., Asahi M., Taniike M. (2004). Cardiac-specific disruption of the c-raf-1 gene induces cardiac dysfunction and apoptosis. J. Clin. Invest..

[46] Kandadi M.R., Hua Y., Ma H., Li Q., Kuo S.R., Frankel A.E., Ren J. (2010). Anthrax lethal toxin suppresses murine cardiomyocyte contractile function and intracellular Ca^2+^ handling via a NADPH oxidase-dependent mechanism. PLoS One.

[47] Cui X., Li Y., Li X., Haley M., Moayeri M., Fitz Y., Leppla S.H., Eichacker P.Q. (2006). Sublethal doses of *Bacillus anthracis* lethal toxin inhibit inflammation with lipopolysaccharide and *Escherichia coli* challenge but have opposite effects on survival. J. Infect. Dis..

[48] Hanna P.C., Acosta D., Collier R.J. (1993). On the role of macrophages in anthrax. Proc. Natl. Acad. Sci. USA.

[49] Tournier J.N., Rossi Paccani S., Quesnel-Hellmann A., Baldari C.T. (2009). Anthrax toxins: A weapon to systematically dismantle the host immune defenses. Mol. Aspects. Med..

[50] Pezard C., Berche P., Mock M. (1991). Contribution of individual toxin components to virulence of *Bacillus anthracis*. Infect. Immun..

[51] Molin F.D., Fasanella A., Simonato M., Garofolo G., Montecucco C., Tonello F. (2008). Ratio of lethal and edema factors in rabbit systemic anthrax. Toxicon.

[52] Quinn C.P., Shone C.C., Turnbull P.C., Melling J. (1988). Purification of anthrax-toxin components by high-performance anion-exchange, gel-filtration and hydrophobic-interaction chromatography. Biochem. J..

[53] Cooksey B.A., Sampey G.C., Pierre J.L., Zhang X., Karwoski J.D., Choi G.H., Laird M.W. (2004). Production of biologically active *Bacillus anthracis* edema factor in *Escherichia coli*. Biotechnol. Prog..

[54] Jernigan J.A., Stephens D.S., Ashford D.A., Omenaca C., Topiel M.S., Galbraith M., Tapper M., Fisk T.L., Zaki S., Popovic T. (2001). Bioterrorism-related inhalational anthrax: The first 10 cases reported in the United States. Emerg. Infect. Dis..

[55] Griffith T.M., Taylor H.J. (1999). Cyclic AMP mediates EDHF-type relaxations of rabbit jugular vein. Biochem. Biophys. Res. Commun..

[56] Stehlik J., Movsesian M.A. (2006). Inhibitors of cyclic nucleotide phosphodiesterase 3 and 5 as therapeutic agents in heart failure. Expert. Opin. Investig. Drugs.

[57] Borer J.S. (2004). Drug insight: If inhibitors as specific heart-rate-reducing agents. Nat. Clin. Pract. Cardiovasc. Med..

[58] Katz A.M. (2001). Physilogy of the Heart.

[59] Shen Y., Zhukovskaya N.L., Zimmer M.I., Soelaiman S., Bergson P., Wang C.R., Gibbs C.S., Tang W.J. (2004). Selective inhibition of anthrax edema factor by adefovir, a drug for chronic hepatitis B virus infection. Proc. Natl. Acad. Sci. USA.

[60] Friedler R.M., Kurokawa K., Coburn J.W., Massry S.G. (1975). Renal action of cholera toxin: I. Effects on urinary excretion of electrolytes and cyclic AMP. Kidney Int..

[61] Kurokawa K., Friedler R.M., Massry S.G. (1975). Renal action of cholera toxin: II. Effects on adenylate cyclase-cyclic AMP system. Kidney Int..

[62] Pierce N.F., Graybill J.R., Kaplan M.M., Bouwman D.L. (1972). Systemic effects of parenteral cholera enterotoxin in dogs. J. Lab. Clin. Med..

[63] Kuehnert M.J., Doyle T.J., Hill H.A., Bridges C.B., Jernigan J.A., Dull P.M., Reissman D.B., Ashford D.A., Jernigan D.B. (2003). Clinical features that discriminate inhalational anthrax from other acute respiratory illnesses. Clin. Infect. Dis..

[64] Firoved A.M., Moayeri M., Wiggins J.F., Shen Y., Tang W.J., Leppla S.H. (2007). Anthrax edema toxin sensitizes DBA/2J mice to lethal toxin. Infect. Immun..

[65] Maldonado-Arocho F.J., Fulcher J.A., Lee B., Bradley K.A. (2006). Anthrax oedema toxin induces anthrax toxin receptor expression in monocyte-derived cells. Mol. Microbiol..

[66] Paccani S.R., Tonello F., Ghittoni R., Natale M., Muraro L., D’Elios M.M., Tang W.J., Montecucco C., Baldari C.T. (2005). Anthrax toxins suppress T lymphocyte activation by disrupting antigen receptor signaling. J. Exp. Med..

[67] Tournier J.N., Quesnel-Hellmann A., Mathieu J., Montecucco C., Tang W.J., Mock M., Vidal D.R., Goossens P.L. (2005). Anthrax edema toxin cooperates with lethal toxin to impair cytokine secretion during infection of dendritic cells. J. Immunol..

[68] Chakrabarty K., Wu W., Booth J.L., Duggan E.S., Nagle N.N., Coggeshall K.M., Metcalf J.P. (2007). Human lung innate immune response to *Bacillus anthracis* spore infection. Infect. Immun..

[69] Heninger S., Drysdale M., Lovchik J., Hutt J., Lipscomb M.F., Koehler T.M., Lyons C.R. (2006). Toxin-deficient mutants of *Bacillus anthracis* are lethal in a murine model for pulmonary anthrax. Infect. Immun..

[70] Pickering A.K., Osorio M., Lee G.M., Grippe V.K., Bray M., Merkel T.J. (2004). Cytokine response to infection with *Bacillus anthracis* spores. Infect. Immun..

[71] Stearns-Kurosawa D.J., Lupu F., Taylor F.B., Kinasewitz G., Kurosawa S. (2006). Sepsis and pathophysiology of anthrax in a nonhuman primate model. Am. J. Pathol..

[72] Popov S.G., Villasmil R., Bernardi J., Grene E., Cardwell J., Popova T., Wu A., Alibek D., Bailey C., Alibek K. (2002). Effect of *Bacillus anthracis* lethal toxin on human peripheral blood mononuclear cells. FEBS Lett..

[73] Triantafilou M., Uddin A., Maher S., Charalambous N., Hamm T.S., Alsumaiti A., Triantafilou K. (2007). Anthrax toxin evades Toll-like receptor recognition, whereas its cell wall components trigger activation via TLR2/6 heterodimers. Cell Microbiol..

[74] Hsu L.C., Ali S.R., McGillivray S., Tseng P.H., Mariathasan S., Humke E.W., Eckmann L., Powell J.J., Nizet V., Dixit V.M. (2008). A NOD2-NALP1 complex mediates caspase-1-dependent IL-1beta secretion in response to *Bacillus anthracis* infection and muramyl dipeptide. Proc. Natl. Acad. Sci. USA.

[75] Cui X., Su J., Li Y., Shiloach J., Solomon S., Kaufman J.B., Mani H., Fitz Y., Weng J., Altaweel L. (2010). *Bacillus anthracis* cell wall produces injurious inflammation but paradoxically decreases the lethality of anthrax lethal toxin in a rat model. Intensiv. Care Med..

[76] Iyer J.K., Khurana T., Langer M., West C.M., Ballard J.D., Metcalf J.P., Merkel T.J., Coggeshall K.M. (2010). Inflammatory cytokine response to *Bacillus anthracis* peptidoglycan requires phagocytosis and lysosomal trafficking. Infect. Immun..

[77] Langer M., Malykhin A., Maeda K., Chakrabarty K., Williamson K.S., Feasley C.L., West C.M., Metcalf J.P., Coggeshall K.M. (2008). *Bacillus anthracis* peptidoglycan stimulates an inflammatory response in monocytes through the p38 mitogen-activated protein kinase pathway. PLoS One.

[78] Popov S.G., Popova T.G., Hopkins S., Weinstein R.S., MacAfee R., Fryxell K.J., Chandhoke V., Bailey C., Alibek K. (2005). Effective antiprotease-antibiotic treatment of experimental anthrax. BMC Infect. Dis..

[79] Adekoya O.A., Sylte I. (2009). The thermolysin family (M4) of enzymes: Therapeutic and biotechnological potential. Chem. Biol. Drug Des..

[80] Fisher J.F., Mobashery S. (2010). Mechanism-based profiling of MMPs. Methods Mol. Biol..

[81] Holty J.E., Bravata D.M., Liu H., Olshen R.A., McDonald K.M., Owens D.K. (2006). Systematic review: A century of inhalational anthrax cases from 1900 to 2005. Ann. Intern. Med..

[82] Migone T.S., Subramanian G.M., Zhong J., Healey L.M., Corey A., Devalaraja M., Lo L., Ullrich S., Zimmerman J., Chen A. (2009). Raxibacumab for the treatment of inhalational anthrax. N. Engl. J. Med..

[83] Centers for Disease Control and Prevention E-IND Protocol: One Time Emergency Use of Liquid 5% Anthrax Immune Globulin for Treatment of Severe Anthrax.

[84] Walsh J.J., Pesik N., Quinn C.P., Urdaneta V., Dykewicz C.A., Boyer A.E., Guarner J., Wilkins P., Norville K.J., Barr J.R. (2007). A case of naturally acquired inhalation anthrax: Clinical care and analyses of anti-protective antigen immunoglobulin G and lethal factor. Clin. Infect. Dis..

[85] Subramanian G.M., Cronin P.W., Poley G., Weinstein A., Stoughton S.M., Zhong J., Ou Y., Zmuda J.F., Osborn B.L., Freimuth W.W. (2005). A phase 1 study of PAmAb, a fully human monoclonal antibody against *Bacillus anthracis* protective antigen, in healthy volunteers. Clin. Infect. Dis..

[86] Altaweel L., Chen Z., Moayeri M., Cui X., Li Y., Su J., Fitz Y., Johnson S., Leppla S.H., Purcell R. (2011). Delayed treatment with W1-mAb, a chimpanzee-derived monoclonal antibody against protective antigen, reduces mortality from challenges with anthrax edema or lethal toxin in rats and with anthrax spores in mice. Crit. Care Med..

